# Primary treatment and relative survival by stage and age in vaginal carcinoma: a population-based SweGCG study

**DOI:** 10.2340/1651-226X.2026.45598

**Published:** 2026-09-30

**Authors:** Kristina Hellman, Maria Bjurberg, Christer Borgfeldt, Pernilla Dahm Kähler, Angelique Flöter Rådestad, Jenny-Maria Jönsson, Preben Kjølhede, Cecilia Olsson, Camilla Sköld, Katja Stenström Bohlin, Karin Stålberg, Erik Holmberg, Elisabeth Åvall Lundqvist

**Affiliations:** a Department of Gynecologic Cancer, Theme Cancer, Karolinska University Hospital, Stockholm, Sweden; b Department of Hematology, Oncology, and Radiation Physics, Skåne University Hospital, Lund University, Lund, Sweden; c Department of Biomedical and Clinical Sciences, Linköping University, Linköping, Sweden; d Clinical Department of Obstetrics and Gynecology in Linköping, Region Östergötland, Linköping, Sweden; e Department of Obstetrics and Gynecology, Sahlgrenska Academy at University of Gothenburg, Gothenburg, Sweden; f Department of Gynecology and Reproductive Medicine, Sahlgrenska University Hospital, Gothenburg, Sweden; g Division of Neonatology, Gynecology and Obstetrics, Department of Women’s and Children’s Health, Karolinska Institutet, Stockholm, Sweden; h Division of Oncology, Department of Clinical Sciences, Lund University, Lund, Sweden; i Department of Health Sciences, Faculty of Health, Science, and Technology, Karlstad University, Karlstad, Sweden; j Department of Bachelor in Nursing, Lovisenberg Diaconal University College, Oslo, Norway; k Department of Immunology, Genetics and Pathology, Cancer Precision Medicine, Uppsala University, Uppsala, Sweden; l Department of Women’s and Children’s Health, Uppsala University, Uppsala, Sweden; m Department of Oncology, Sahlgrenska Academy at University of Gothenburg, Gothenburg, Sweden

**Keywords:** vaginal carcinoma, stage, age, treatment, survival

## Abstract

**Background and purpose:**

This study aimed to investigate treatment patterns and survival outcomes in population-based data describing treatment and survival of primary vaginal carcinoma (VC) in a routine clinical setting, stratified by age group and stage.

**Methods:**

This nationwide, population-based study included women diagnosed with epithelial VC between 2011 and 2020, registered in the Swedish Quality Register for Gynecologic Cancer. Five-year relative survival (RS) was estimated using the Pohar Perme method. The excess mortality rate ratio (EMRR) was assessed by Poisson regression.

**Results:**

In total, 252 women were identified, 29% were aged ≥ 80 years. The median follow-up time was 7.5 years. The most common stages were I and II (26% each). Definitive radiotherapy, with or without chemotherapy, was administered to 59% of patients. Among women with stage I, 48% underwent primary surgery and 29% received adjuvant radiotherapy. Treatment patterns differed across age groups. Among women aged ≥ 80 years, staging was omitted in 18%, and in stage I disease 25% received palliative therapy. None of the irradiated women aged ≥ 80 years received chemoradiotherapy, compared to all women aged 20–59 years. The overall 5-year RS for stages I–IV was 51% (95% confidence interval [CI] 43–58). In stages I–II, the 5-year RS was 28% among women aged ≥80 years and 84% among those aged < 60 years (EMRR of 5.2; 95% CI 1.7–15.6).

**Interpretation:**

Primary treatment of vaginal cancer in Sweden generally followed international guidelines, except among women aged ≥ 80 years. The markedly poorer RS in this age group suggests both diagnostic and therapeutic compromises.

## Introduction

Primary vaginal carcinoma (VC) is a rare malignancy arising in the vagina, but without involvement of the cervix or vulva, and it accounts for approximately 2% of all gynecologic cancers [1]. In Sweden, the age-standardized incidence rate was 0.6 per 100,000 person-years in 2022 and has remained stable since 1960 [2]. The median age at diagnosis is 70 years [3]. Histologically, squamous cell carcinoma is the most common subtype (≈85%), followed by adenocarcinoma (10–15%) [1]. The risk factors for VC are similar to those for cervical carcinoma and include Human papilloma virus (HPV) infection (present in approximately 50–70% of cases), smoking, and immunosuppression.

Due to its rarity, there are no randomized clinical trials addressing treatment, and most published studies are retrospective and involve a limited number of women. As a result, current treatment guidelines rely largely on retrospective data and expert opinion rather than high-level evidence [4, 5] and management strategies are often extrapolated from treatment protocols for cervical cancer. In Sweden, regional guidelines were replaced by the first national guideline for VC in 2016 [6], which are in line with the recently published international guidelines (National Comprehensive Cancer Network [NCCN] and European Society of Gynaecological Oncology [ESGO]) [4, 5].

According to most studies, radiotherapy is considered the standard treatment across all stages of disease [7, 8]. In early-stage disease, though, surgical management may offer outcomes comparable to those of radiotherapy [8, 9]. Chemoradiotherapy (CRT) and brachytherapy have been associated with improved survival and local control [7, 10, 11].

Five-year age-standardized relative survival (RS) for VC has remained relatively low over the past decades in Europe: 40% for women diagnosed 2000–2007 [12], and 45% for those diagnosed 2010–2015 [13]. Previous studies indicate that older women have poorer prognosis compared to younger women [3, 14] but data are lacking whether treatment patterns vary between different age-groups.

In this nationwide population-based study, we aimed to examine treatment patterns and survival outcomes stratified by stage and age-group among women diagnosed with primary VC in Sweden between 2011 and 2020.

## Material and methods

### Study population

The Swedish Quality Register for Gynecologic Cancer (SQRGC) was used to identify eligible women. Inclusion criteria were age ≥18 years, histologically verified primary VC (International Classification of Diseases ([ICD]-10 code C52) diagnosed and treated in Sweden from January 1st, 2011 to December 31th, 2020.

Data extracted from the SQRGC database included date of diagnosis, age, International Federation of Gynecology and Obstetrics (FIGO) stage, histology, details on primary treatment, treatment intention and vital status. Stage X corresponds to unknown or undetermined stage reflecting that staging was not possible because the required diagnostic procedures, including imaging or biopsy, were not performed.

Tumor classification was performed according to the World Health Organization (WHO) standards [15]. The classification by FIGO 2009 was used for staging [1]. Treatment recommendations were based on Swedish Guidelines for VC, which were adapted from FIGO recommendations [16].

### Swedish Quality Registry for Gynecologic Cancer

The Swedish National Cancer Registry (NCR) was founded in 1958 and covers over 95% of malignant tumors in Sweden [17]. Registration to NCR is mandatory but only persons that have official residency (and hence a personal identification number) in Sweden are included in the cancer register. In the NCR, clinical data regarding treatment and follow-up are not registered, therefore the SQRGC was established, previously described in detail [18]. Reporting to the SQRGC is done electronically and prospectively by all hospitals and clinics in the six healthcare regions in Sweden. Data from the register can be achieved using the personal identification number, which is linked to both the NCR and the National Causes of Death Registry, allowing for coverage control and long-term patient follow-up. VC has been registered in the SQRGC since 2011. The coverage rate of primary VC in the SQRGC, compared to NCR, has varied between 91 and 100% from 2012 to 2023 [19]. The validity of the SQRGC data has recently been investigated, showing 70–100% agreement between registered data and the original data in medical records [20]. Women are notified about being included in the quality registry at diagnosis. Consent is assumed unless the patient objects, and withdrawal can be made at any time.

## Statistics

The women were classified into three age-groups: < 60 years, 60–79 years, and ≥ 80 years to determine the impact of age on survival.

Survival time was defined as the interval from the date of diagnosis until the date of death, date of emigration, or last time of follow-up (September 7th, 2023) whichever occurred first. Median follow-up time, with interquartile range (IQR), is reported for the entire study cohort and separately for participants who were alive at the end of follow-up.

Overall survival (OS) and RS with 95% confidence intervals (CIs) were estimated. OS was calculated using Kaplan–Meier estimates [21] and survival curves were compared with the log-rank test. Univariable and multivariable Cox proportional hazards models [22] were used to estimate hazard ratios (HRs) with 95% CIs for overall mortality, while Poisson regression models were applied to estimate excess mortality rate ratios (EMRRs) with 95% CIs. The proportional hazards assumption was assessed using Schoenfeld residuals [23]. RS interpreted as a measure of net survival (i.e. survival from the disease of interest in the absence of other causes of death), was estimated using the Pohar–Perme estimator [24] implemented in Stata via the stns command [25]. Graphical presentation of RS curves was also generated with stns. Expected survival, stratified by attained age, sex, and calendar year, was derived from national life tables provided by Statistics Sweden. For regression modeling of RS and multivariable analyses, the Stata strs command [26] was applied. All statistical analyses were performed using Stata (version 19.5, StataNow/MP for Mac; StataCorp LLC, College Station, TX, USA). A two-sided *p*-value <0.05 was considered statistically significant.

## Results

In total, 279 women with primary vaginal cancer were identified in the SQRGC. Non-epithelial cancers (*n* = 27) were excluded (melanoma, *n* = 19 and sarcoma, *n* = 8), thus 252 women with primary epithelial VC constituted the study group. The median follow-up time for the entire cohort was 3.4 years (IQR 1.1–7.1) and 7.5 years (IQR 5.0–9.9) for participants who were alive at the end of follow-up.

The median age of women at diagnosis was 71 years (min–max, 26–88 years), and 29% were ≥ 80 years (Table 1). Most cancers (84%) consisted of squamous cell carcinoma and 10% of adenocarcinomas. Half of the women (52%) were diagnosed in FIGO stages I and II (Table 1). Women aged < 60 years were more frequently allocated stage I (36%) than higher stages whereas women ≥ 80 years were allocated stage I and stage II in 22%, respectively, and undetermined stage (X) in 18%, see Table 1. Women classified as stage X had a median age of 85 years.

**Table 1:** FIGO stage, histology and primary treatment of women with primary vaginal carcinoma in relation to age groups.

Variable	20–59 years	60–79 years	80–100 years	Total
	*N* = 59 *n* (%)	*N* = 120 *n* (%)	*N* = 73 *n* (%)	*N* = 252 *n* (%)
**FIGO stage** ^**a**^				
I	21 (36)	28 (23)	16 (22)	65 (26)
II	11 (19)	40 (33)	16 (22)	67 (26)
III	12 (20)	24 (20)	11 (15)	47 (19)
IVA	4 (7)	10 (8)	8 (11)	22 (9)
IVB	9 (15)	12 (10)	9 (12)	30 (12)
X	2 (3)	6 (5)	13 (18)	21 (8)
**Histology**				
Squamous cell carcinoma	44 (75)	107 (89)	62 (85)	213 (84)
Adenocarcinoma	12 (20)	8 (7)	6 (8)	26 (10)
Other^b^	3 (5)	5 (4)	5 (7)	13 (5)
**Primary treatment**				
Surgery	14 (24)	25 (21)	8 (11)	47 (19)
Surgery alone	10 (71)	11 (44)	5 (56)	26 (54)
Postoperative ART	4 (28)	14 (56)	3 (33)	21 (46)
*EBRT*	1 (25)	9 (64)	3 (100)	13 (62)
*Chemoradiotherapy*	3 (75)	5 (36)	0 (0)	8 (38)
Definitive radiotherapy, DRT	34 (58)	78 (65)	36 (49)	148 (59)
*EBRT*	6 (18)	35 (45)	36 (100)	77 (52)
*Chemoradiotherapy*	28 (82)	43 (55)	0 (0)	71 (48)
NACT as part of treatment	1 (2)	3 (2)	0 (0)	4 (2)
Other combinations^c^	8 (14)	0 (0)	1 (1)	9 (4)
Chemotherapy alone	2 (3)	6 (5)	2 (3)	10 (4)
No antitumoral treatment	0 (0)	6 (5)	22 (30)	28 (11)
*Missing*	0 (0)	2 (2)	4 (6)	6 (2)
**Treatment intention**				
Curative	53 (90)	95 (79)	26 (36)	174 (69)
Palliative	3 (5)	16 (13)	36 (49)	55 (22)
Not assessable	3 (5)	8 (7)	9 (12)	20 (8)
*Missing*	0	1	2	3

ART: adjuvant radiotherapy; EBRT: external beam radiotherapy; NACT: neoadjuvant chemotherapy.

a FIGO stage according to the 2009 classification. Stage X = undetermined stage.

b Adenosquamous carcinoma (*n* = 5), undifferentiated carcinoma (*n* = 6), serous carcinoma (*n* = 2).

c Surgery + other combinations (*n* = 3), chemoradiotherapy/EBRT + other combinations (*n* = 3), other combinations (*n* = 4).

The most common treatment for VC was definitive radiotherapy (DRT), with or without CRT, while primary surgery was a less frequent treatment (59 vs. 19%). The CRT was used in 48% of women receiving DRT. In women receiving postoperative adjuvant radiotherapy (ART), 38% received CRT. Antitumoral treatment was omitted in 30% of women ≥ 80 years compared to 5% among those aged 60–79 years. All women < 60 years received antitumoral treatment. The treatment intention varied with age-group and was curative in 69% of all women, varying from 90% among women < 60 years to 36% among women ≥ 80 years (Table 1).

In stage I, surgery was performed in nearly half (48%) of the women and postoperative ART was provided to 29%, see Table 2. The frequency of both surgery and ART, though, varied between age groups, see Table 3. Surgery was less common among women ≥ 80 years old compared to those <60 years (31 vs. 48%). The proportion of women in stage I who received CRT, either as ART or DRT, also decreased with age-group from 100% in women < 60 years old, to 33 and 40%, respectively, in age group 60–79 years, and to 0% in the oldest group ≥ 80 years old (Table 3).

**Table 2:** Primary treatment by FIGO stage in women with primary vaginal carcinoma.

Variable	FIGO stage						
	I	II	III	IVA	IVB	X	Total
	*N* = 65 *n* (%)	*N* = 67 *n* (%)	*N* = 47 *n* (%)	*N* = 22 *n* (%)	*N* = 30 *n* (%)	*N* = 21 *n* (%)	*N* = 252 *n* (%)
**Age** (median, min–max)	68 (26–93)	70 (36–88)	73 (33–99)	74 (48–94)	67 (27–91)	85 (45–95)	71 (26–88)
**Primary Treatment**							
Surgery	31 (48)	8 (12)	0 (0)	2 (9)	2 (7)	4 (19)	47 (19)
Surgery alone	22 (71)	1 (12)	0 (0)	0 (0)	0 (0)	3 (75)	26 (55)
Postoperative ART	9 (29)	7 (88)	0 (0)	2 (100)	2 (100)	1 (25)	21 (45)
*EBRT*	5 (56)	4 (57)	0 (0)	1 (50)	2 (100)	1 (100)	13 (62)
*Chemoradiotherapy*	4 (44)	3 (43)	0 (0)	1 (50)	0 (0)	0 (0)	8 (38)
DRT	27 (42)	53 (79)	37 (79)	15 (68)	11 (37)	5 (24)	148 (59)
*EBRT*	14 (52)	26 (49)	21 (57)	9 (60)	6 (54)	1 (20)	77 (52)
*Chemoradiotherapy*	13 (48)	27 (51)	16 (43)	6 (40)	5 (46)	4 (80)	71 (48)
NACT as part of treatment	1 (1)	0 (0)	1 (2)	0 (0)	1 (3)	1 (5)	4 (2)
Other combinations^a^	3 (5)	1 (1)	2 (4)	0 (0)	3 (10)	0 (0)	9 (4)
Chemotherapy alone	0 (0)	0 (0)	3 (6)	0 (0)	6 (20)	1 (5)	10 (4)
No antitumoral treatment	2 (3)	5 (8)	3 (6)	4 (18)	6 (20)	8 (38)	28 (11)
*Missing*	1	0	1	1	1	2	6
**Treatment intention**							
Curative	58 (89)	58 (87)	30 (64)	9 (41)	11 (37)	8 (38)	174 (69)
Palliative	6 (9)	4 (6)	12 (26)	11 (50)	14 (47)	8 (38)	55 (22)
Not assessable	1 (2)	5 (8)	4 (8)	2 (9)	5 (17)	3 (14)	20 (8)
*Missing*	0	0	1	0	0	2	3

IQR: interquartile range; ART: adjuvant radiotherapy; EBRT: external beam radiotherapy; DRT: definitive radiotherapy; NACT: neoadjuvant chemotherapy.

a Surgery + other combinations (*n* = 3), chemoradiotherapy/EBRT + other combinations (*n* = 3), other combinations (*n* = 4).

**Table 3:** Treatment in FIGO stage I by age-groups in women with primary vaginal carcinoma.

Variable	20–59 years	60–79 years	80–100 years	Total
	*N* = 21 *n* (%)	*N* = 28 *n* (%)	*N* = 16 *n* (%)	*N* = 65 *n* (%)
**Primary treatment**				
Surgery	10 (48)	16 (57)	5 (31)	31 (48)
Surgery only	8 (80)	10 (62)	4 (80)	22 (71)
Postoperative ART	2 (20)	6 (38)	1 (20)	9 (29)
*EBRT*	0 (0)	4 (67)	1 (100)	5 (55)
*Chemoradiotherapy*	2 (100)	2 (33)	0 (0)	4 (44)
Definitive radiotherapy	9 (43)	10 (36)	8 (50)	27 (42)
*EBRT*	0 (0)	6 (60)	8 (100)	14 (52)
*Chemoradiotherapy*	9 (100)	4 (40)	0 (0)	13 (48)
NACT as part of treatment	0 (0)	1 (4)	0 (0)	1 (2)
Other combinations^a^	2 (10)	0 (0)	1 (6)	3 (5)
No antitumoral treatment	0 (0)	1 (4)	1 (6)	2 (3)
*Missing*	0	0	1	1
**Treatment intention**				
Curative	21 (100)	26 (93)	11 (69)	58 (89)
Palliative	0 (0)	2 (7)	4 (25)	6 (9)
Not assessable	0 (0)	0 (0)	1 (6)	1 (2)

ART: adjuvant radiotherapy; EBRT: external beam radiotherapy; NACT: neoadjuvant chemotherapy.

a Surgery + other combinations (*n* = 1), chemoradiotherapy/EBRT + other combinations (*n* = 1), other combinations (*n* = 1).

Most women in stage II–IVA (77%) were treated with DRT, with the use of combined CRT varying between 40 and 51% (Table 2 and Supplement Table 1). In stage IVA, 18% did not receive any antitumoral treatment. For women in stage IVB, treatment consisted of DRT (37%), chemotherapy alone (20%) or no treatment at all (20%). In stage X 38% of the women received no antitumoral treatment.

### Survival outcome

The 5-year OS and RS for the total study population including stage X was 45% (95% CI 39–52) and 49% (95% CI 42–56), respectively (Table 4)*.* The corresponding survival rates excluding stage X were 47% (95% CI 40–53) and 51% (95% CI 43–58), respectively (Table 4).

**Table 4:** Five-year overall survival (OS), relative survival (RS), and results from univariable Cox proportional hazards regression (hazard ratio, HR) and univariable Poisson regression of excess mortality rate (EMRR) among women with vaginal carcinoma, stratified by age group and FIGO stage.

FIGO stage^a^	Age-group years	Patients (*n*)	OS (%) (95% CI)	HR (95% CI)	*P*	RS (%) (95% CI)	EMRR (95% CI)	*P*
All stages (I–IV, X^b^):	20–59	59	68.8 (55.0–79.1)	Reference		69.4 (55.3–80.0)	Reference	
	60–79	120	55.2 (45.8–63.7)	1.59 (0.93–2.71)	0.089	59.7 (49.3–68.7)	1.45 (0.83–2.53)	0.197
	≥ 80	73	11.8 (5.0–19.8)	5.39 (3.19–9.13)	< 0.001	15.1 (6.2–27.7)	4.42 (2.52–7.78)	< 0.001
	All	252	45.5 (39.2–51.6)			49.1 (41.7–56.4)		
Stages I–IV	All	231	47.1 (40.4–53.5)			50.8 (43.2–58.5)		
Stage I–II:	20–59	32	83.5 (64.7–92.8)	Reference		84.3 (71.2–97.5)	Reference	
	60–79	68	62.5 (49.7–72.9)	2.71 (1.04–7.09)	0.042	67.6 (55.0–80.1)	2.48 (0.90–6.85)	0.080
	≥ 80	32	20.8 (8.7–36.4)	7.58 (2.89–19.9)	< 0.001	28.3 (8.3–48.2)	5.17 (1.72–15.6)	0.003
	All	132	57.2 (48.1–65.2)			61.7 (51.4–72.1)		
Stage III–IV:	20–59	25	47.3 (26.9–65.2)	Reference		47.8 (28.3–67.2)	Reference	
	60–79	46	45.3 (30.6–59.0)	1.12 (0.57–2.19)	0.735	49.1 (33.7–64.5)	1.05 (0.53–2.09)	0.892
	≥ 80	28	3.6 (0.2–15.4)	4.50 (2.29–8.84)	< 0.001	6.2 (0.0–15.9)	4.18 (2.06–8.46)	< 0.001
	All	99	34.0 (24.8–43.4)			36.5 (26.1–47.0)		

a FIGO staging classification 2009.

b Stage X: undetermined stage.

The 5-year RS for stages I, II, III, IV were: 67, 57, 36 and 20%, respectively, see Figure 1A and Table S2. Figure 1B shows the 5-year RS (15%) for the age-group ≥ 80 years compared to those younger than 60 years (69%), cf. Table 4. The 5-year RS for the oldest age-group, ≥ 80 years, was significantly decreased in both FIGO stage I–II (28.3%; 95% CI 8.3–48.2, EMRR 5.2; 95% CI 1.72–15.6, *p* = 0.003) as well as in FIGO stage III–IV (6.2%; 95% CI 0.0–15.9, EMRR 4.2; 95% CI 2.1–8.46, *p* < 0.001) compared to women 20–59 years (Figure 1C and D, Table 4).

**Figure 1:**
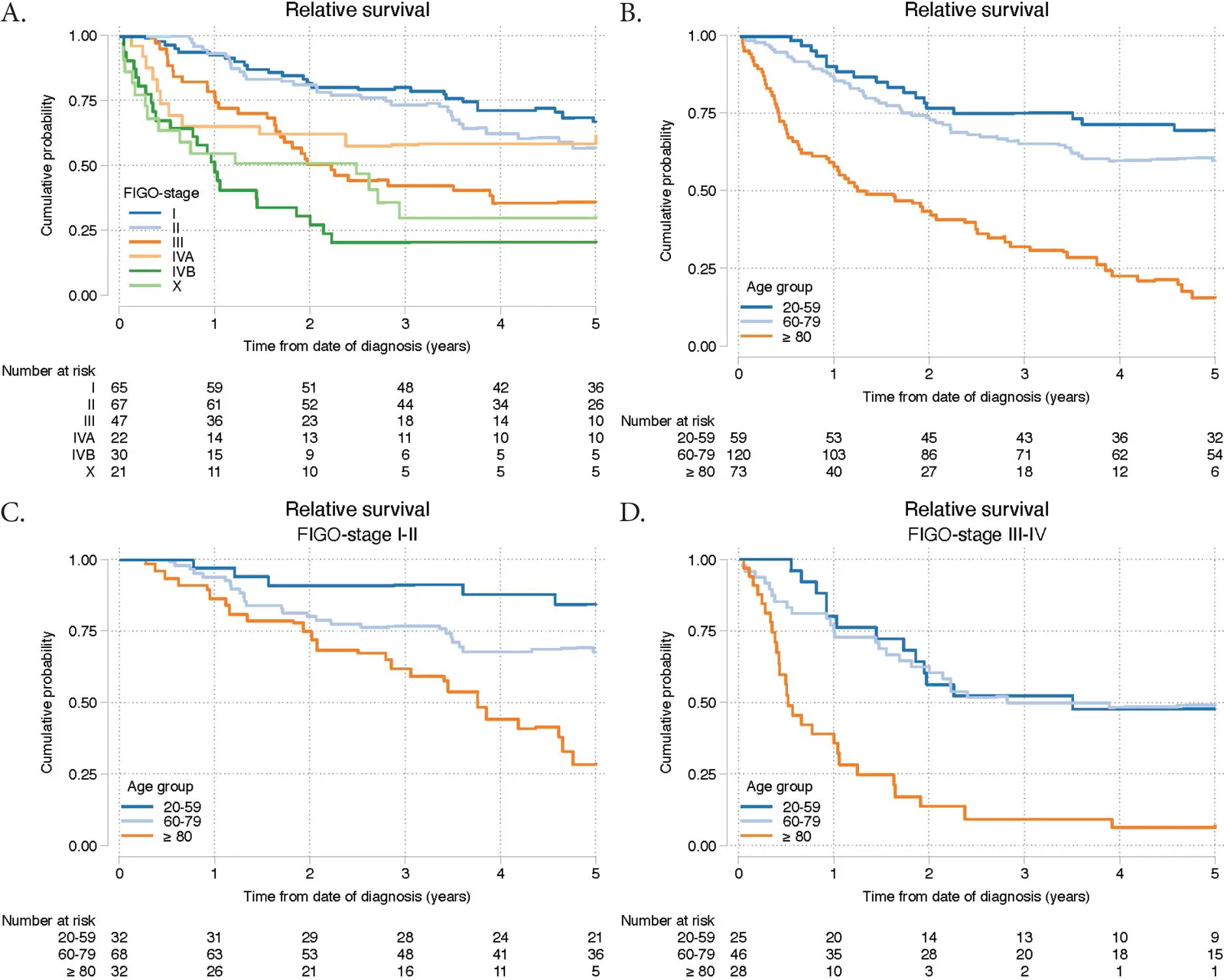
(A–D) Relative survival of women diagnosed with vaginal carcinoma 2011–2020 in Sweden by FIGO stage and age-group (stage X included).

Further survival analyses included only VC patients treated with curative intent. In stage I, there was no OS difference between women treated with primary surgery (*n* = 30), with or without ART, compared to DRT (*n* = 25) with or without CRT (5-year OS 76%; 95% CI 56–88 vs. 54%; 95% CI 32–71, *p* = 0.13). In stage I–IV, the 5-year OS was significantly better for women treated with definitive CRT (*n* = 70) compared to radiotherapy alone (*n* = 53), (5-year OS 72%; 95% CI 60–81 vs. 39%; 95% CI 26–52, *p* < 0.001), Figure 2.

**Figure 2:**
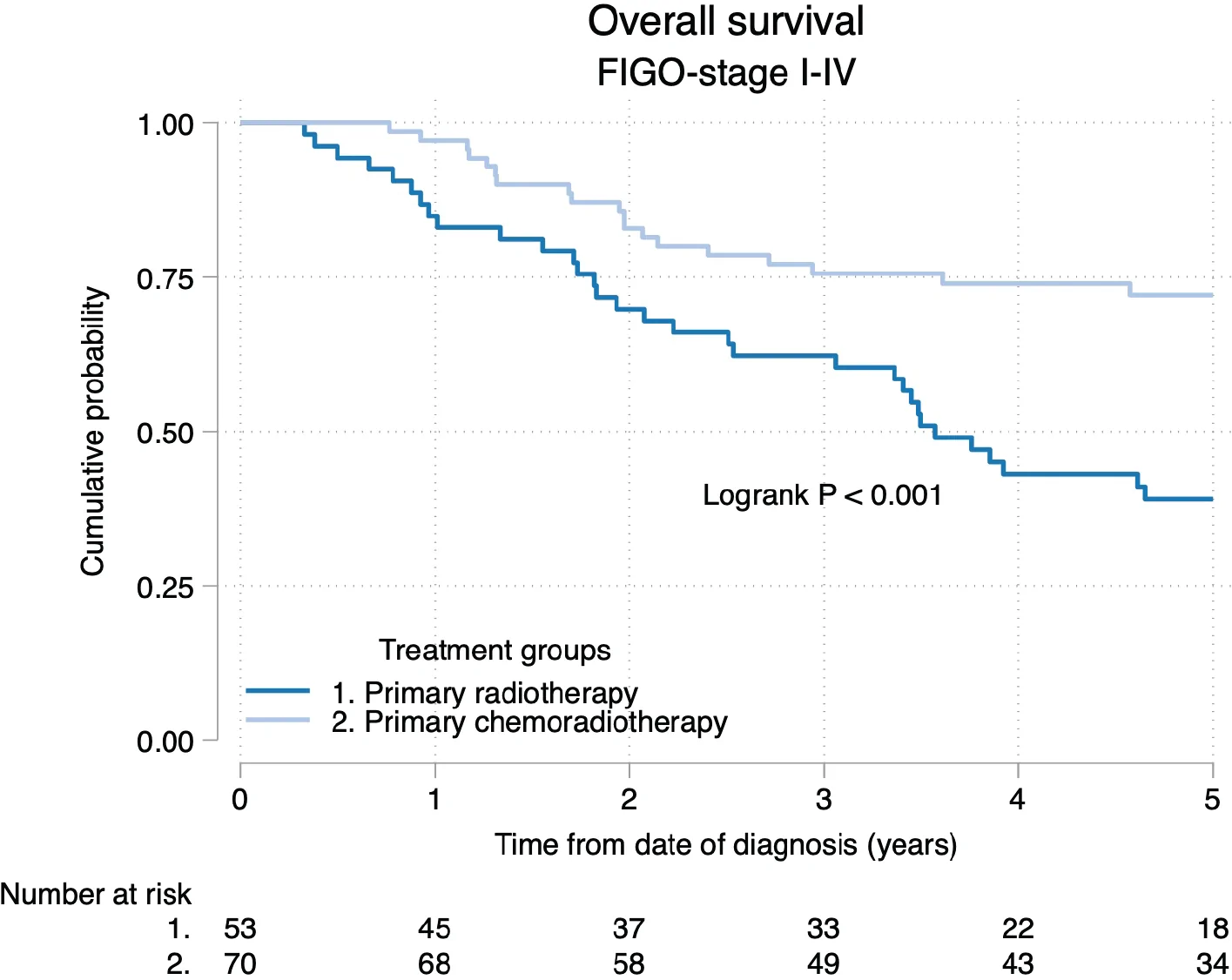
Overall survival of patients diagnosed with vaginal carcinoma treated with curative radiotherapy or concomitant chemoradiotherapy.

Furthermore, the prognostic impact of the covariates DRT with or without concomitant chemotherapy, age-group, FIGO stage and histology among women with VC treated with curative DRT (*n* = 123) was investigated. In the univariable analysis, only definitive CRT (*p* < 0.001) and age-group <80 years (*p* = 0.005) were associated with OS (Table S3). In the multivariable analysis, definitive CRT was the only independent predictor for increased survival (HR 0.46, 95% CI 0.24–0.90, *p* = 0.024).

## Discussion

To our knowledge, no other unselected population-based published study with nearly a complete nationwide coverage, and strong validity, has provided such detailed information on primary treatment patterns and survival outcomes in primary VC. The most important finding in our study was that treatment patterns varied between age groups, also in early-stage disease, and older women aged ≥ 80 years had significantly poorer RS than younger women in stage I disease.

DRT was the most common primary treatment, but no woman ≥ 80 years of age received concomitant CRT whereas all irradiated women aged 20–59 did. Most women were diagnosed with early-stage VC. However, among women aged ≥ 80 years nearly one fifth had an undetermined stage, indicating incomplete staging procedures. As female life expectancy is projected to increase (in Sweden from 85 years in 2024 to 90 years by 2070) the need for appropriate management of elderly women will become increasingly important [27].

### Treatment patterns

The primary treatment patterns in all stages for women with VC in our study were consistent with previously published reports [9, 14, 28, 29]. In stage I, the recommended treatment according to current European guidelines [5] is either surgery or DRT, with or without CRT and brachytherapy, depending on tumor size and localization. In our cohort, nearly half of the women in stage I underwent surgery, which is a high proportion compared to 26–36% in previously published studies [9, 14, 28, 29]. However, the proportion of women receiving ART was lower, compared to 45–82% reported in earlier studies [9, 29–31], suggesting an appropriate patient selection. This aligns with current guidelines [5] that recommend avoiding the combination of surgery and radiotherapy due to the increased risk of morbidity, an approach supported by evidence extrapolated from cervical cancer studies [1, 5].

Women aged ≥ 80 years with stage I disease were more frequently selected for DRT, possibly due to more extensive tumor size at diagnosis or the presence of comorbidities rendering them unsuitable for surgery. Large tumor size (>2–4 cm) is an independent negative prognostic factor [10, 32], and may contribute to poorer survival even in stage I disease. Other independent prognostic factors included in the multivariable analyses were age [10, 32], stage [10, 32], histology, treatment, year of diagnosis [10] and competing nonsignificant factors were marital status, race, pathological grade [10], cervical dysplasia, asymptomatic or not, lymph node metastases at diagnosis, growth in the rectovaginal septum, and tumor longitudinal and circumferential location [32]. As the SQRGC does not include data on tumor size we were unable to determine whether older women had larger tumors at diagnosis, which could potentially contribute to the observed survival differences in stage I.

Definitive platinum-based CRT combined with brachytherapy is currently the preferred treatment modality for FIGO stage II–IVA vaginal cancer [4, 5] and previous studies report that 56–84% of patients undergo DRT. This is in line with our study of VC in whom 68–79% received DRT, while 40–51% underwent CRT. The study period (2011–2020) encompasses the transition period from radiotherapy alone to CRT, which likely contributed to the relatively low proportion of patients receiving CRT.

None of the women ≥ 80 years received CRT, either as ART or as part of DRT. CRT in vaginal cancer is recommended as ART or as part of DRT extrapolated from randomized trials in cervical cancer [33, 34]. The first large-scale study (*n* = 8086) demonstrating a survival benefit of CRT compared to radiotherapy alone in vaginal cancer was published in 2014 [11], followed by a subsequent Surveillance, Epidemiology, and End Results (SEER) database analysis in 2020 [10]. In line with these findings, our study confirmed CRT as an independent predictor of improved OS among women treated with DRT, regardless of stage or patient age. However, the use of CRT decreased with advancing age.

Consistent with our results, two previously published studies both based on SEER data have reported that women aged ≥65 years and ≥ 80 years, respectively, were less likely to receive CRT compared to younger women [10, 35]. However, in one of the studies only patients 65 years or older and stages II–IVA were included [35]. The reduced use of CRT in older women may reflect concerns regarding increased treatment-related toxicity and an uncertainty considering the benefit of CRT for elderly patients. We are not aware of any randomized controlled trials comparing DRT and definitive CRT among elderly patients in various age-groups but published retrospective studies in cervical cancer report concurrent CRT in elderly to be associated with improved outcomes similar as in younger counterparts without increasing serious toxicities [36, 37]. The benefit and tolerability of CRT by elderly patients have also been reported in other malignancies e.g. glioblastoma and gastrointestinal cancers [38, 39]. Elderly glioblastoma patients with Karnofsky performance status > 70 and Charlson Comorbidity Index (CCI) < 3 are therefore suggested to be enrolled in combined prospective studies [38]. The published data in other malignancies indicate that combined CRT for elderly women with few comorbidities and good performance status may provide a survival benefit without increasing the risk for serious morbidity hence we suggest that CRT should also be considered for elderly women with primary VC.

In this population, cisplatin can be replaced with carboplatin, a less toxic alternative that has been demonstrated to be safe and tolerable in older patients [40, 41].

### Survival

The reported 5-year RS for vaginal cancer in previous population-based studies has ranged between 38 and 68% [3, 13, 28, 42, 43], which is consistent with the findings of our study. As expected, survival decreased significantly with increasing FIGO stage. Recently, data from the German nationwide registry including 2194 women with VC reported an overall 5-year RS of 58% (95% CI 55–61%) [3]. The corresponding 5-year RS for early stages was 82% (95 CI 77–86%) for stage I and 59% (95% CI 52–66%) for stage II, which exceed our estimates of 67% (95% CI 52–81%) and 57% (95% CI 42–71%), respectively, however, the CIs overlap. The direct comparisons should also be interpreted with caution due to the smaller sample size in our study and the absence of information on therapeutic management and treatment intent in the study by Forner et al. [3]. In addition, 45% of the patients were excluded due to no information on tumor stage at initial diagnosis in their study [3]. In the survival analyses including the entire population (*n* = 252), we included women treated with palliative intent as well as those receiving no antitumoral treatment. When restricting the analysis to women treated with curative CRT in FIGO stages I–IV, a 5-year OS of 72% was observed.

We observed a pronounced decline in RS with increasing age, with women ≥ 80 years exhibiting more than a fourfold higher risk of excess mortality compared to younger age groups. Even among those with early-stage disease, RS was significantly lower in the oldest age group. Several previous studies have similarly demonstrated a negative association between age and survival [3, 10, 13, 14, 29, 35]. However, only one prior study has evaluated survival across age groups stratified by stage. In a SEER based analysis including 1781 women diagnosed 2004–2014, women aged ≥ 80 years had a significantly higher risk of death across all stages compared to those aged 18–49 years, with a HR of 3.62 for stage I disease [14], consistent with our findings. However, in contrast to our study they had excluded patients with no Tumor, Nodes, Metastasis (TNM) stage and had no information on treatment intentions.

Poorer survival in older women may reflect less aggressive treatment, potentially influenced by comorbidities and age-related factors. As the disease primarily affects older women, curative treatment should not be withheld based on chrono-logical age alone. Instead, decisions should be individualized according to biological age and functional status. Although some patients may be best managed with supportive care, many older women can tolerate and benefit from active treatment. Therefore, broader health assessments, including geriatric evaluation, may help guide management and improve outcomes [44].

The key strength of our study is the unselected, population-based cohort with real world data covering more than 90% of the Swedish population. In comparison, the SEER database includes less than 30% of the United States population. Another strength lies in the high validity of the SQRGC, which has demonstrated excellent concordance between registered data and original medical records [20]. An additional advantage is the SQRGC’s linkage to other national health databases, ensuring accurate information on vital status for women with cancer. A limitation of our study is the small sample size, restricting the statistical analyses that could be performed. Other limitations include the lack of detailed information on radiotherapy parameters, such as radiation doses, treatment duration, and use of brachytherapy. These factors are important determinants of prognosis and could have provided insight into whether older patients received suboptimal or palliative treatment, as well as whether prolonged treatment times due to interruptions may have influenced outcomes. Another limitation is that the SQRGC does not include data on comorbidities, performance status or tumor size. Data on comorbidities and performance status could have helped explain treatment decisions, including the choice between curative and palliative treatment or between surgery and radiotherapy. Furthermore, as tumor size is an established independent prognostic factor, its inclusion might have contributed to a better understanding of the ob-served differences in treatment strategies and survival among older patients.

In conclusion, this nationwide population-based study demonstrates that treatment patterns for primary vaginal cancer in Sweden largely adhere to guidelines, except for women aged 80 years or older. The study further highlights the persistently low 5-year survival rates, particularly among elderly patients. The results emphasize the urgent need for the development and implementation of novel treatment strategies. Moreover, the integration of frailty assessments into clinical decision-making may facilitate more individualized and effective treatment planning, ensuring that older women receive care that is both appropriate and potentially life-prolonging.

## Supplementary Material


