# Supplementary material for: Primary treatment and relative survival by stage and age in vaginal carcinoma: a population-based SweGCG study
Source: Acta Oncol. 2026 Sep 30;65:45598. doi: 10.2340/1651-226X.2026.45598 (PMC13635840; doi:10.2340/1651-226X.2026.45598)
Supplement: Supplementary file 1 [file AO-65-45598-s1.pdf]

Supplementary material has been published as submitted. It has not been copyedited, or typeset by Acta Oncologica

Supplementary tables

Table S1. Treatment per age-group by FIGO stages II-IVA in women with primary vaginal carcinoma.

Variable	20-59 years N=27 n (%)	60-79 years N=74 n (%)	80-100 years N=35 n (%)	Total N=136 n (%)
**Primary Treatment**				
Surgery	2 (7)	7(9)	1 (3)	10 (7)
Surgery only	1 (50)	0 (0)	0 (0)	1 (10)
Postoperative ART	1(50)	7 (100)	1 (100)	9 (90)
EBRT	0 (0)	4 (57)	1 (100)	5 (56)
Chemoradiotherapy	1 (100)	3 (43)	0 (0)	4 (44)
Definitive Radiotherapy, DRT	22 (81)	60 (81)	23 (66)	105 (77)
EBRT	5 (23)	28 (47)	23(100)	56 (53)
Chemoradiotherapy	17 (77)	32 (53)	0 (0)	49 (47)
NACT as part of treatment	0 (0)	1 (1)	0 (0)	1 (1)
Other combinations[a]	3 (11)	0 (0)	0 (0)	3 (2)
Chemotherapy only	0 (0)	1 (1)	2 (18)	3 (2)
No antitumoral treatment	0 (0)	4 (5)	8 (23)	12 (9)
Missing	0	1	1	2
**Treatment intention**				
Curative	25 (93)	58 (78)	14 (40)	97 (71)
Palliative	1 (4)	8 (11)	18 (51)	27 (4)
Not assessable	1 (4)	7 (9)	3 (9)	11 (8)
Missing	0	1	0	1

ART, adjuvant radiotherapy; EBRT, external beam radiotherapy; NACT, neoadjuvant chemotherapy.

[a] Surgery + other combinations

Table S2. The 5-year overall survival (OS), relative survival (RS), hazard ratios and relative risk of excess mortality (EMRR) of women with vaginal carcinoma by FIGO stage.

FIGO stage[a]	Patients (n)	OS (%) (95% CI)	HR (95% CI)	P	RS (%) (95% CI)	EMRR (95% CI)	P
I	65	62.4 (49.3-73.0)	Reference		66.8 (52.4-81.2)	Reference	
II	67	52.0 (39.0-63.4)	1.32 (0.77-2.25)	0.311	56.7 (42.2-71.3)	1.26 (0.64-2.48)	0.496
III	47	33.6 (20.6-47.1)	2.44 (1.43-4.16)	0.001	35.9 (21.1-50.6)	2.81 (1.50-5.28)	0.001
IVA	22	54.6 (32.1-72.4)	1.66 (0.79-3.47)	0.179	58.3 (34.7-81.9)	1.69 (0.68-4.21)	0.258
IVB	30	20.0 (8.1-35.6)	4.68 (2.64-8.27)	<0.001	20.4 (6.3-34.5)	5.58 (2.90-10.7)	<0.001
X[b]	21	27.8 (10.9-47.7)			29.7 (7.9-51.5)		

[a]FIGO staging classification 2009. [b]Stage X: undetermined stage.

Table S3. Uni- and multivariable Cox regression analyses on overall survival among women with primary vaginal carcinoma treated with curative definitive radiotherapy (n=123).

		Univariable		Multivariable[a]	
Variable	n	HR (95%CI)	P	HR (95%CI)	P
**Treatment**					
Radiotherapy alone	53	1.0		1.0	
Chemoradiotherapy	19/70 (27%)	0.38 (0.22-0.67)	0.001	0.46 (0.24-0.90)	0.024
**Age group*,** years					
20-59	8/32 (24%)	1.0			
60-79	29/71 (41%)	1.90 (0.87-4.15)	0.110	1.53 (0.68-3.44)	0.304
80-100	14/19 (74%)	3.43 (1.44-8.19)	0.005	1.83 (0.67-5.00)	0.241
**FIGO stage***					
I-II	32/75 (43%)	1.0			
III-IV	17/44 (39%)	1.00 (0.55-1.79)	0.987		
Missing	2/4 (50%)				
**Histology**					
SCC	44/109 (40%)	1.0			
Other	7/14 (50%)	1.20 (0.54-2.67)	0.649		

*The proportional hazards assumption was not met, and the estimated hazard ratios (HRs) should therefore be interpreted as average effects over the follow-up period.

[a] Only significant covariates from the univariable analysis were included in the multivariable model. Age-group had a confounding effect on treatment, however not significant

SCC, squamous cell carcinoma
